# Intracellular zinc signaling via Krüppel-like transcription factor 6 promotes HuD expression in pancreatic β cell

**DOI:** 10.1016/j.gendis.2023.101144

**Published:** 2023-10-16

**Authors:** Seongho Cha, Chongtae Kim, Myeongwoo Jung, Seungyeon Ryu, Sukyoung Han, Wook Kim, Eun Kyung Lee

**Affiliations:** a Department of Biochemistry; Department of Biomedicine & Health Sciences, College of Medicine, The Catholic University of Korea, Seoul 06591, South Korea; b Catholic Institute for Visual Science, College of Medicine, The Catholic University of Korea, Seoul 06591, South Korea; c Department of Molecular Science & Technology, Ajou University, Suwon 16499, South Korea

Dysfunction of pancreatic β cells caused by zinc deficiency is related to the pathogenesis of diabetes.^1^ Impaired zinc homeostasis in diabetes is associated with reduced zinc transporters.^2^ Down-regulation of HuD, an essential factor for normal β cell function, has been shown in diabetes.^3^ To assess the correlation between cellular zinc level and HuD expression in diabetes, relative levels of HuD and ZIP8, a highly expressed Zrt-, Irt-like protein (ZIP) transporter protein in β cells,^4^ were analyzed between *db/db* mice and control wild-type mice. HuD and ZIP8 expressions were down-regulated in the pancreas of *db/db* mice compared with that in control mice (Fig. 1A, B). Cellular zinc content was also reduced in pancreatic islets of *db/db* mice (Fig. 1B). These results suggest a positive correlation between intracellular zinc contents and HuD expression in the islet of the pancreas.

**Figure 1:**
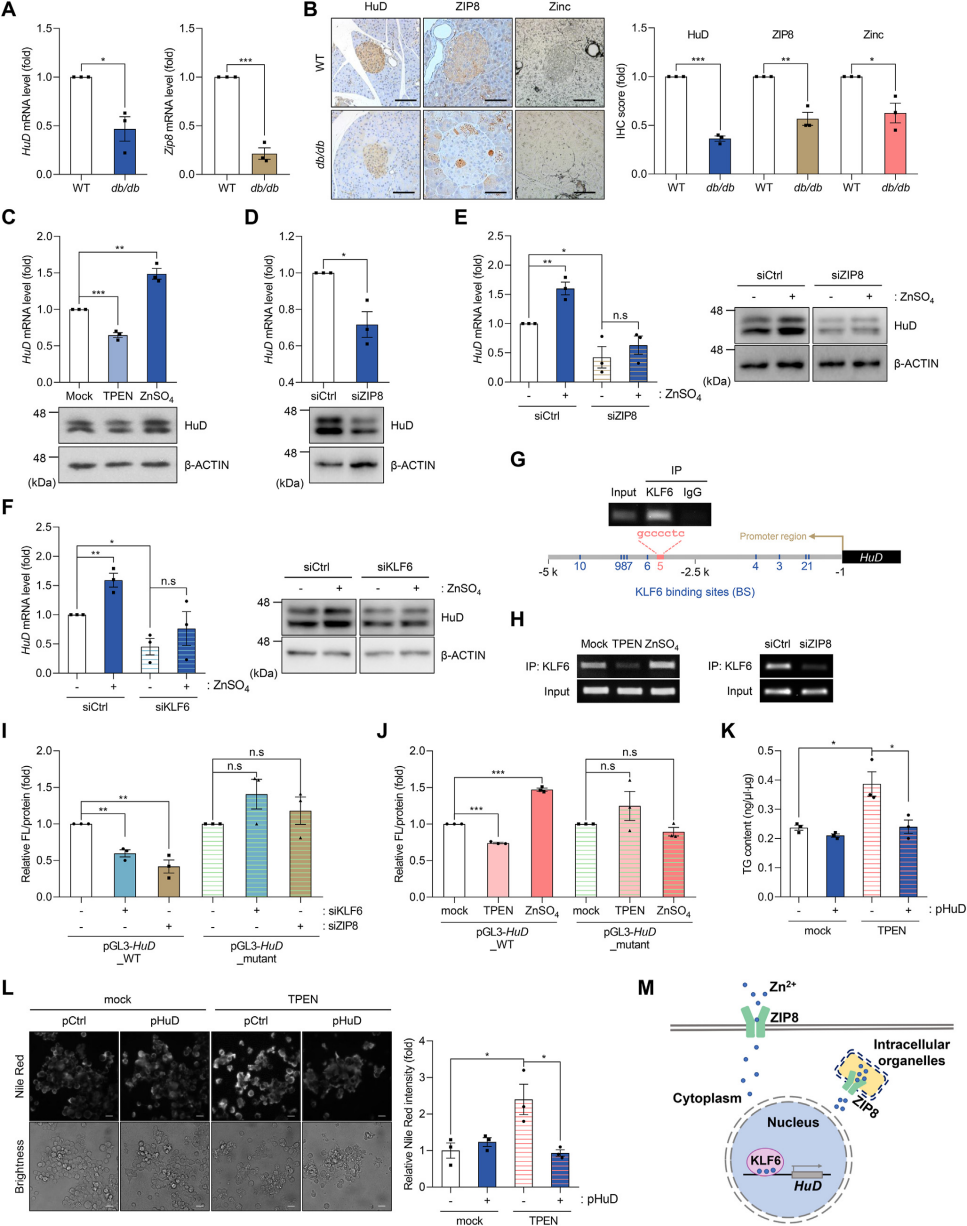
Zinc/ZIP8/KLF6-mediated regulation of HuD expression in pancreatic β cells. **(A)** After isolating total RNAs from *db/db* mice and age-matched WT mice (C57BLKS/J) (*n* = 3), levels of *HuD* and *Zip8* mRNAs were determined by RT-qPCR. **(B)** Protein levels of HuD and ZIP8 were analyzed by immunohistochemistry and cellular zinc contents in paraffin-embedded pancreatic tissues were assessed by Timm staining. Densitometric analysis of each image (*n* = 3) was done using ImageJ software. Scale bar, 100 μm. **(C–F)** βTC6 cells were incubated with TPEN (2 μM) or ZnSO_4_ (100 μM) for 72 h (C) or transfection with siCtrl or siZIP8 for 48 h (D). After being transfected with siZIP8 (E) or siKLF6 (F), βTC6 cells were further incubated with ZnSO_4_ (100 μM). *HuD* mRNA and HuD protein levels were determined by RT-qPCR and western blotting analysis, respectively. **(G)** Association between KLF6 and the BS5 area (−3156 ∼ −2956 nt region) in the upstream region of the *HuD* gene was analyzed by chromatin immunoprecipitation (ChIP) assay followed by PCR using a specific primer set D (Fig. S5B). **(H)** After treatment or knockdown of ZIP8, the relative binding of KLF6 to the BS5 area was assessed by ChIP-PCR. Amplified DNA fragments by PCR were analyzed by gel electrophoresis. **(I, J)** Reporter analysis. After transfection of siRNAs (I) or treatment (J), βTC6 cells were sequentially transfected with the reporter plasmids. Relative reporter expression was determined by luciferase assay. **(K, L)** After HuD-overexpressing plasmid (pHuD) transfection, βTC6 cells were further incubated with TPEN for 72 h to deplete cellular zinc. Cellular triglyceride (TG) content was evaluated with a triglyceride assay kit (K) and intracellular lipid droplets were detected by fluorescence microscopy via Nile Red staining (L). Fluorescent signals were quantified using ImageJ software. Scale bar, 20 μm. **(M)** Schematic model of zinc/ZIP8/KLF6-mediated HuD expression. *Gapdh* mRNA was used as a reference gene for normalization of RT-qPCR results and β-ACTIN was used as a loading control for western blotting. Images are representative and data are presented as mean ± SEM of three independent analyses. Statistical significance of data was analyzed via Student's *t*-test; n.s., not significant (*P* > 0.05); ^∗^*P* < 0.05, ^∗∗^*P* < 0.01, ^∗∗∗^*P* < 0.001.

To investigate whether cellular zinc level could affect HuD expression in pancreatic β cells, HuD expression in mouse insulinoma βTC6 cells maintained in 25 mM of glucose/DMEM was analyzed after modulating intracellular zinc concentration by depletion (with a zinc-chelator TPEN), supplementation (with a nutrient supplement zinc sulfate (ZnSO_4_)), or knockdown of ZIP8. Cellular zinc level in β cells was visualized with a fluorescence microscope using a cell-permeable dye FluoZin™-3 (Fig. S1A and B). Zinc depletion caused a decrease in HuD expression. In contrast, zinc supplementation reversed it (Fig. 1C). In addition, *ZIP8* knockdown down-regulated HuD level (Fig. 1D). However, the levels of other Hu family proteins, including *HuR*, *HuB*, and *HuC*, were not significantly changed by cellular zinc level (Fig. S2). Additionally, *ZIP8* knockdown alleviated ZnSO_4_-mediated HuD induction, as shown in Figure 1E. These results suggest that cellular zinc level and ZIP8 regulate HuD expression in pancreatic β cells.

To understand the downstream pathway of zinc-mediated transcriptional regulation of the *HuD* gene, putative transcription factors responsible for HuD expression were explored by searching zinc-dependent transcription factors with binding sites near the upstream regions of the *HuD* gene. *In silico* analysis using the TRANSFAC® database identified Krüppel-like transcription factor 6 (KLF6) as one of the putative transcription factors responsible for HuD expression. Its putative binding sites near the transcription start site of the *HuD* gene were predicted in Figure S3. To validate whether KLF6 could regulate HuD expression, the levels of HuD were assessed after transfecting βTC6 cells with siRNA against the *KLF6* gene. *KLF6* knockdown down-regulated HuD expression (Fig. S4) and interfered with ZnSO_4_-mediated HuD induction (Fig. 1F). These results suggest that KLF6 is involved in the zinc-mediated regulation of HuD expression in pancreatic β cells.

To understand the mechanism of KLF6-mediated HuD expression, the interaction between KLF6 and *HuD* gene was investigated by chromatin immunoprecipitation assay, followed by PCR. As shown in Figure S5A, the upstream region of the *HuD* gene (up to ∼−5 kb) has several putative binding sites for KLF6. Each binding between KLF6 and *HuD* gene was experimentally validated using specific primer sets (Fig. S5B). KLF6 binding was observed only in the binding site 5 (BS5) region of the upstream region of the *HuD* gene that was amplified with primer set D (Fig. 1G). Based on this observation, the relative KLF6 binding to the BS5 region was analyzed after zinc depletion or supplementation. Zinc depletion with TPEN decreased the binding of KLF6 to the BS5 region, while zinc supplementation with ZnSO_4_ increased it compared with the control group (Fig. 1H). In addition, *ZIP8* knockdown decreased the association between KLF6 and the BS5 region of the *HuD* gene (Fig. 1H). To examine whether KLF6 could regulate the transcription of *HuD*, luciferase reporter vectors (pGL3-*HuD*_WT and pGL3-*HuD*_Mutant) were generated by cloning the DNA fragments including the upstream region of the *HuD* gene (−3115 ∼ −1 bp region from transcription start site) or the same region with BS5 sequence mutated (Fig. S6). After knockdown or treatment in reporter-transfected cells, relative luciferase activity in each group was determined by luciferase assay. Knockdown of *ZIP8* or *KLF6* decreased luciferase activity in the pGL3-HuD_WT transfected cells, but not in the mutant group (Fig. 1I). Zinc depletion or supplementation also affected reporter expression as observed in Figure 1I (Fig. 1J). These results suggest that the signaling via zinc, ZIP8, and KLF6 regulates HuD expression.

HuD has been shown to regulate intracellular triglyceride (TG) accumulation in pancreatic β cells.^5^ To investigate the effect of zinc depletion on the TG content of β cells, cellular TG levels were assessed after TPEN treatment. Zinc depletion increased TG content in βTC6 cells (Fig. 1K). However, ectopic expression of HuD reversed TPEN-induced TG accumulation (Fig. 1K, L). These results indicate that zinc depletion increases TG content via HuD down-regulation and that HuD overexpression can reverse TPEN-induced intracellular lipid accumulation in β cells.

In summary, we proposed a novel regulatory mechanism of HuD expression via zinc signaling in pancreatic β cells (Fig. 1M) to provide insight into understanding the molecular action of zinc supplementation. Our results indicated that zinc signaling could promote HuD expression by activating its transcription via a zinc finger transcription factor KLF6. While zinc deficiency can lead to β cell dysfunction in the pathogenesis of diabetes, zinc supplementation or HuD expression could help restore normal β cell function. These results suggest that the zinc/KLF6/HuD axis is essential for normal β cell functions. Further studies are needed to determine whether HuD regulation via zinc signaling is common across different species and whether HuD restoration in β cells could be a valuable strategy for preventing and treating diabetes caused by β cell dysfunction.

## Ethics declaration

All animal experiment procedures were approved by the animal research ethics committee of the Catholic University of Korea (CUMC-2022-0223-01) and examined under the guidelines of the Catholic University of Korea on the Use and Care of Animals.

## Author contributions

S.C., C.K., E.K.L., and W.K. performed study concept and design; S.C. and C.K. performed experiments, analysis and interpretation of data, and statistical analysis; M.J., S.R., and S.H. provided technical support; W.K. and E.K.L wrote and revised the manuscript. All authors read and approved the final manuscript.

## Conflict of interests

The authors have no conflict of interests to declare.

## Funding

This work was supported by the Basic Science Research Programs through the 10.13039/501100003725National Research Foundation of Korea (10.13039/501100003725NRF) grant funded by the Korean government (10.13039/501100014188MSIT) (No. 2021R1A2C1004128).
