# Supplementary material for: Intracellular zinc signaling via Krüppel-like transcription factor 6 promotes HuD expression in pancreatic β cell
Source: Genes Dis. 2023 Oct 16;11(4):101144. doi: 10.1016/j.gendis.2023.101144 (PMC10907158; doi:10.1016/j.gendis.2023.101144)
Supplement: Multimedia component 1 [file mmc1.docx]

**Intracellular zinc signaling via Krüppel-like transcription factor 6 promotes HuD expression in pancreatic β cells**

​Seongho Cha^1, 2, †^, Chongtae Kim^3†^, Myeongwoo Jung^1, 2^, Seungyeon Ryu^1, 2^, Sukyoung Han^1, 2^, Wook Kim^4^, and Eun Kyung Lee^1, 2,^ *

^1^ Department of Biochemistry, ^2^ Department of Biomedicine & Health Sciences, ^3^ Catholic Institute for Visual Science, College of Medicine, The Catholic University of Korea, Seoul 06591, South Korea

^4^ Department of Molecular Science & Technology, Ajou University, Suwon 16499, South Korea

^†^ These authors contributed equally to this work.

* Correspondence should be addressed to Eun Kyung Lee; [leeek@catholic.ac.kr](mailto:leeek@catholic.ac.kr)


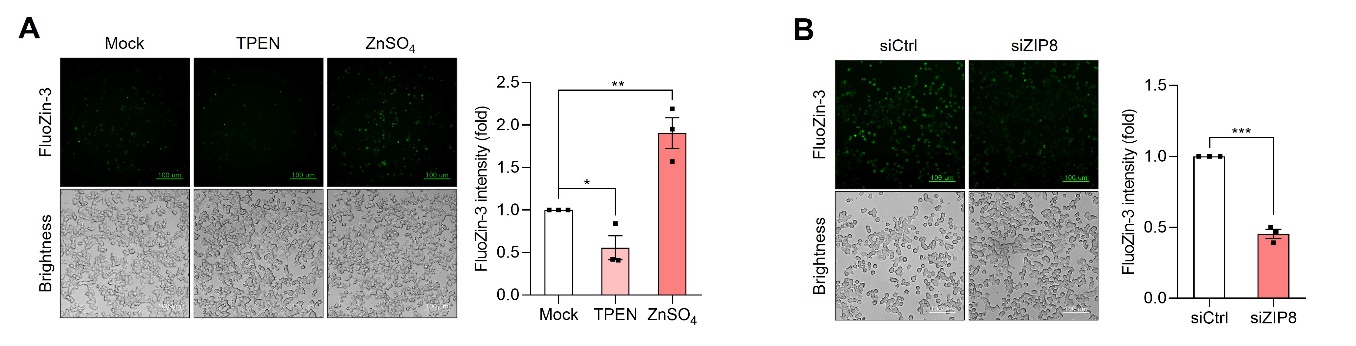


**Supplementary Figure S1. Assessment of cellular zinc levels in pancreatic β cells**

βTC6 cells were incubated with TPEN (2 μM) or ZnSO_4_ (100 μM) for 72 h (A) and transfected with siCtrl or siZIP8 for 48 h (B). For analysis of intracellular zinc level, cells were incubated with a fluorescent dye FluoZin-3 and fluorescent signals were quantified using ImageJ software. Images are representative and data are presented as mean ± SEM of three independent analyses. Scale bar, 100 μm. Statistical significance of data was analyzed via Student’s t-test; *, *p* < 0.05, **, *p* < 0.01.


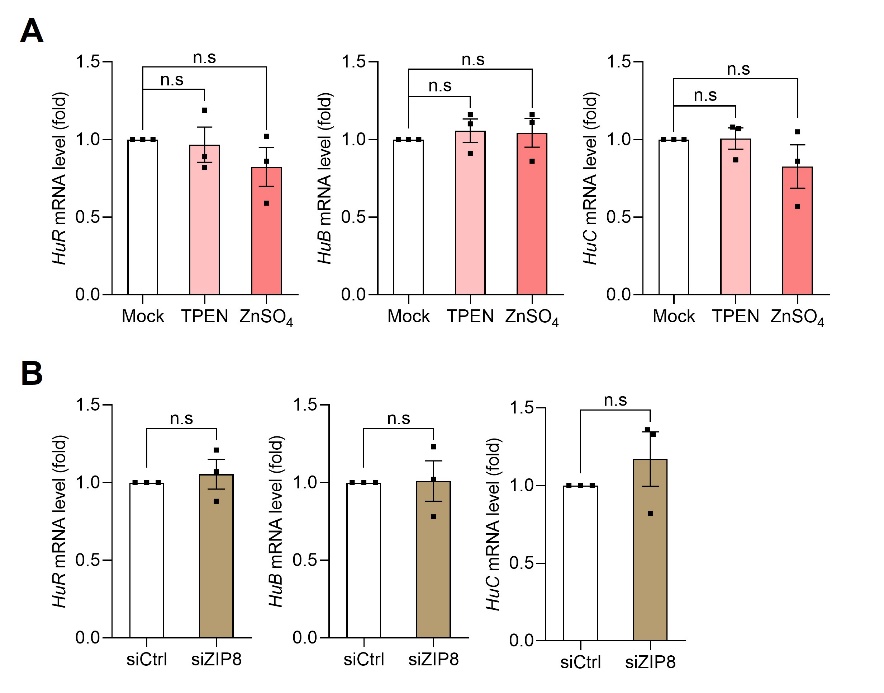


**Supplementary Figure S2. Relative levels of *HuR*, *HuB*, and *HuC* in βTC6 cells after zinc depletion, supplementation, or ZIP8 knockdown**

After incubating βTC6 cells with TPEN (2 μM) or ZnSO_4_ (100 μM) for 72 h (A) or transfecting cells with siCtrl or siZIP8 for 48 h (B), the level of each mRNA was assessed by RT-qPCR. *Gapdh* mRNA was used as a reference gene for RT-qPCR. Data are presented as mean ± SEM of three independent analyses. Statistical significance of data was analyzed via Student’s t-test; n.s, not significant (*p* > 0.05).


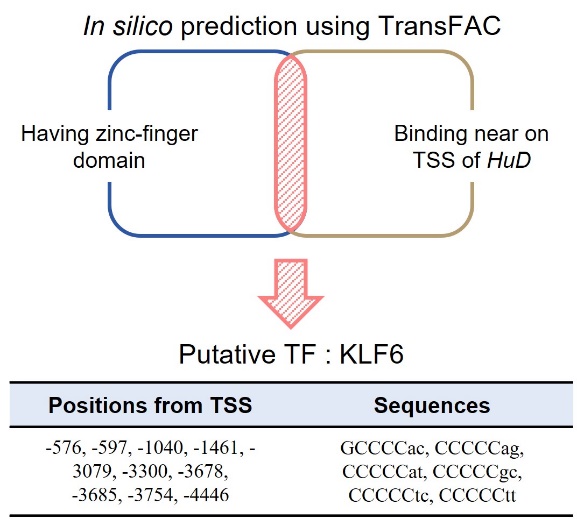


**Supplementary Figure S3. Identification of KLF6 as a novel regulator of HuD expression**

*In silico* analysis using the TransFAC database (<http://www.gene-regulation.com/pub/databases.html>) identified KLF6 as a putative transcription factor that might be responsible for the regulation of *HuD* expression. Putative KLF6-binding positions and sequences on the upstream region of the *HuD* gene transcription start site (TSS) are listed.


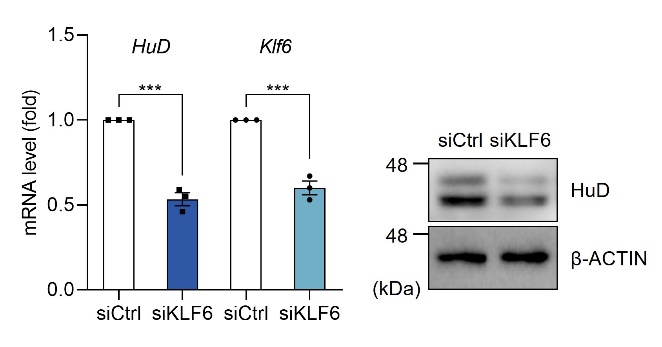


**Supplementary Figure S4. Regulation of HuD expression by KLF6**

After transfection of βTC6 cells with siRNAs, HuD expression was assessed by RT-qPCR and western blotting analysis. *Gapdh* mRNA was used as a reference gene for normalization and β-ACTIN was used as a loading control for WB. Images are representative and data are presented as mean ± SEM of three independent analyses. Statistical significance of data was analyzed via Student’s t-test; ***, *p* < 0.001.


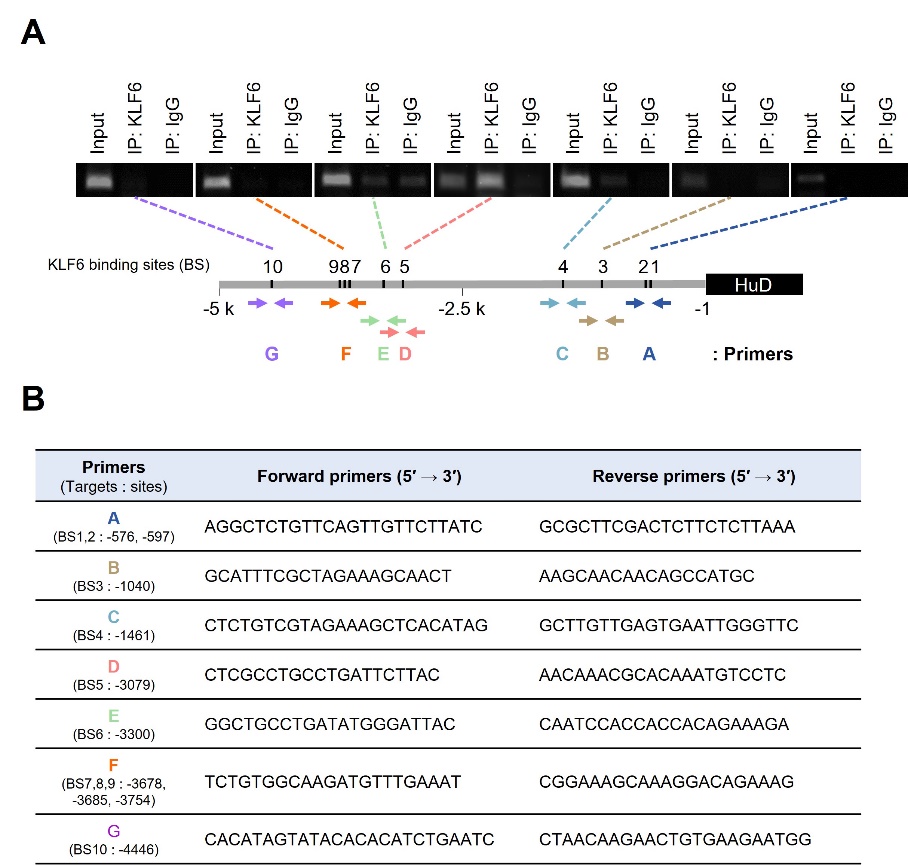


**Supplementary Figure S5. Interactions between KLF6 and the upstream region of the *HuD* gene**

(A) Schematic diagram of the 5′ upstream region of *HuD* gene and experimental validation of KLF6 binding to putative binding sites. The association between KLF6 and *HuD* gene was assessed by ChIP assay followed by PCR. (B) Primer sequences used for KLF6 ChIP-PCR analysis.


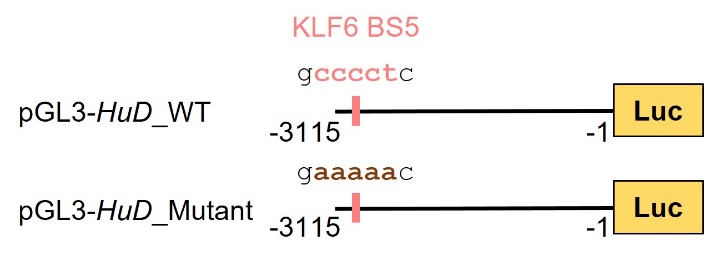


**Supplementary Figure S6. Schematic diagram of reporter constructs**

pGL3-*HuD*_WT was cloned by inserting -3115 ~ -1 nt in the upstream region of the *HuD* gene into a pGL3-Basic plasmid, and pGL3-*HuD*_Mut was generated by site-directed mutagenesis by replacing the sequence of ‘cccct’ in the BS5 (-3079 to -3073 nt; gcccctc) to ‘aaaaa’.

**Supplementary Table S1. Oligonucleotide sequences used in this study**

| **For RT-qPCR** | **Forward primers (5ʹ → 3ʹ)** | **Reverse primers (5ʹ → 3ʹ)** |
| --- | --- | --- |
| *HuR* | GGATGACATTGGGAGAACGAAT | TGTCCTGCTACTTTATCCCGAAT |
| *HuB* | CTGCTCCTCACCAGTTGACTC | ATCTCGCCAATGCTCCCAAAA |
| *HuC* | ATGGTCACTCAGATACTGGGG | TTCTGGGGTAGGTAGTTGACG |
| *HuD* | GCCTCAGGTGTCAAATGGACC | CCATACCCTAAACTCTGTCCTGT |
| *Zip8* | GAACAATTGCCTGGATGATCACGC | AAGCCGGTTAACATCCCTGCATTC |
| *Klf6* | GTTTCTGCTCGGACTCCTGAT | TTCCTGGAAGATGCTACACATTG |
| *Gapdh* | AGGTCGGTGTGAACGGATTTG | TGTAGACCATGTAGTTGAGGTCA |
| **For cloning** | **Forward primers (5ʹ → 3ʹ)** | **Reverse primers (5ʹ → 3ʹ)** |
| pGL3-*HuD*_WT | AAAAGGTACCTGCTTTACAAATGTCACACCACT | AAAACCATGGTCCTAACCCTGCTGCATCCA |
| pGL3-*HuD*_Mutant | TGTAGCGGTCAGAAAAACACGCTGAAAGA | TCTTTCAGCGTGTTTTTCTGACCGCTACA |
| siRNA | **Sense sequences (5ʹ → 3ʹ)** | **Antisense sequences (5ʹ → 3ʹ)** |
| Control siRNA (siCtrl) | AAUUCUCCGAACGUGUCACGUUU | ACGUGACACGUUCGGAGAAUUU |
| siZIP8 #1 | GGGACUAGCUUUCGGCAUUUU | AAUGCCGAAAGCUAGUCCCUU |
| siZIP8 #2 | ACGCAGGAGACAUCGAAUUUU | AAUUCGAUGUCUCCUGCGUUU |
| siZIP8 #3 | GUAAGAAAGCACAACGCAAUU | UUGCGUUGUGCUUUCUUACUU |
| siZIP8 #4 | AGGAAUGAGCACCCGGCAAUU | UUGCCGGGUGCUCAUUCCUUU |
| siKLF6 | CACACAGGAGAAAAGCCUUACAGAUUU | AUCUGUAAGGCUUUUCUCCUGUGUGUU |

**Supplementary Table S2. List of antibodies used in this study**

| **Antibody** | **Company** | **Cat. No.** |
| --- | --- | --- |
| HuD | SantaCruz Biotechnology, Inc. | sc-28299 |
| ZIP8 | Proteintech | 20459-1-AP |
| KLF6 | SantaCruz Biotechnology, Inc. | sc-365633 |
| β-ACTIN | GeneTex, Inc. | GTX629630 |
| Mouse IgG (HRP Conjugated) | Sigma-Aldrich | AP124P |
| Rabbit IgG (HRP Conjugated) | Sigma-Aldrich | AP132P |
| Normal rabbit IgG | SantaCruz Biotechnology, Inc. | sc-2027 |

**Materials & methods**

**Cell culture, transfection, and treatment**

Mouse insulinoma βTC6 cells were purchased from American Type Culture Collection (ATCC) and cultured in Dulbecco’s modified Eagle’s medium (DMEM) containing 25 mM of glucose (Capricorn Scientific, Ebsdorfergrund, Germany) supplemented with 10% fetal bovine serum (FBS) and 1% antibiotics at 37℃, according to the instruction of American Type Culture Collection (ATCC) (https://www.atcc.org/products/crl-3605). Transfection of small interfering RNAs (Genolution Pharmaceuticals, Inc., Seoul, South Korea), plasmids, and reporter clones were done using Lipofectamine^TM^ 2000 (Invitrogen^TM^, Waltham, MA, USA) according to the manufacturer’s instructions. Cells were incubated with 2 μM of N, N, N′, N′-tetrakis (2-pyridylmethyl) ethylenediamine (TPEN) for zinc depletion or 100 μM of zinc sulfate (ZnSO_4_) (Sigma-Aldrich, Burlington, MA, USA) for zinc supplementation.

**RNA analysis**

Total RNAs were isolated from whole cells or mouse pancreas using RNAiso Plus (Takara Bio, Inc., Shiga, Japan). They were used to synthesize cDNAs using the ReverTra^®^ Ace qPCR RT kit (Toyobo Co., Ltd, Osaka, Japan). Relative levels of mRNAs were measured by quantitative PCR (qPCR) using SensiFAST™ SYBR Hi-ROX kit (Meridian Bioscience, Inc., Cincinnati, OH, USA), gene-specific primers (Supplementary Table S1), and StepOnePlus™ Real-Time PCR System (Applied Biosystems™, Waltham, MA, USA). Data were processed using the _ΔΔ_CT method to compare control and experimental groups. *Gapdh* mRNA was used as a reference gene to normalize qPCR results.

**Western blotting**

Whole-cell lysates were prepared using RIPA buffer (Biosesang, Inc., Seongnam, South Korea) containing 1× protease inhibitor cocktail (Roche, Basel, Switzerland). Proteins in cell lysates were then separated by SDS-PAGE and transferred onto polyvinylidene difluoride (PVDF) membranes (Millipore, Burlington, MA, USA). These membranes were incubated with primary antibodies against HuD (Santa Cruz Biotechnology, Inc.) or β-ACTIN (Genetex, Inc., Irvine, CA, USA) at 4℃ overnight and further incubated with horseradish peroxidase (HRP)-conjugated secondary antibodies (Sigma-Aldrich, Burlington, MA, USA). Chemiluminescence was detected by adding a Clarity Western ECL Substrate (Bio-Rad, Inc., Hercules, CA, USA) to the membrane, and images were captured using ChemiDoc Imaging Systems (Bio-Rad, Inc.).

**Animal study**

All animal experiment procedures were approved by the animal research ethics committee of the Catholic University of Korea (CUMC-2022-0223-01) and examined under the guidelines of the Catholic University of Korea on the Use and Care of Animals.

**Immunohistochemistry, Timm staining, and triglyceride assay**

Tissues were fixed in 4% paraformaldehyde, immersed in 20% sucrose, and sectioned at a thickness of 5 µm using a Cryocut Microtome (Leica Biosystems, Germany). After antigen unmasking, slides were blocked with ImmPRESS^®^ HRP Universal (Horse Anti-Mouse/Rabbit IgG) PLUS Polymer Kit, Peroxidase (Vector Laboratories, Inc., Newark, CA, USA) and incubated with anti-HuD or anti-ZIP8 (Proteintech, Chicago, IL, USA) antibodies. Slides were sequentially incubated with anti-Mouse/Rabbit IgG-conjugated Peroxidase (Vector Laboratories) and reacted with 3,3́-diaminobenzidine (DAB) (Agilent, Santa Clara, CA, USA). Signals were analyzed using a panoramic MIDI slide scanner system (3D Histech Ltd., Budapest, Hungary).

Cellular zinc contents in the tissues were assessed by Timm staining using an FD Rapid Timmstain^TM^ kit (FD Neurotechnologies, Columbia, MD, USA) according to the manufacturer’s protocols. Images were taken using an Axioimager M1 microscope (Carl Zeiss Gottingen, Germany). Densitometric analysis was performed using Image J (<https://imagej.net/ij/index.html>) ^1^ and Immunohistochemistry Image Analysis Toolbox (<https://imagej.nih.gov/ij/plugins/ihc-toolbox/index.html>).

Intracellular triglyceride (TG) contents were determined using a Triglyceride Quantification kit (Sigma-Aldrich, Burlington, MA, USA) according to the manufacturer’s instructions. After incubating cell lysates with the reagent for hydrolysis, the absorbance of each sample was measured at 540 nm using a BioTex Synergy HTX (Agilent, Santa Clara, CA, USA).

**Fluorescence microscopy**

Cells were incubated with 1 µM of Fluozin^TM^-3, AM (Invitrogen^TM^, Waltham, MA, USA) or 0.2 µg/mL of Nile red (Sigma-Aldrich, Burlington, MA, USA) for detecting intracellular zinc or lipid droplet, respectively. Fluorescent signals were acquired using a fluorescence IX71 microscope (Olympus, Tokyo, Japan) and quantified using ImageJ software.

**Luciferase assay**

The luciferase reporter plasmid pGL3-*HuD*_WT was generated by cloning the upstream region of the mouse *HuD* gene (-3115 ~ -1 nt region) into a pGL3-Basic vector (Promega, Madison, WI, USA). A mutant reporter plasmid (pGL3-*HuD*_Mutant) was produced via site-directed mutagenesis by substituting the sequence of ‘cccct’ in the KLF6 binding site BS5 (-3079 to -3073 nt; gcccctc) to ‘aaaaa’. After transfection or treatment, cells were sequentially transfected with the reporter plasmids. Luciferase reporter expression was then assessed using a Dual-Luciferase^®^ reporter assay system (Promega, Madison, WI, USA). Luminescent signals were measured and normalized with the protein amount of each sample.

**Chromatin immunoprecipitation (ChIP) assay**

Cells were incubated with 1% formaldehyde, lysed with RIPA buffer, and sonicated with Biorupture^®^ (Cosmo bio. Co., LTD, Tokyo, Japan) to fragment chromatin. After diluting the lysates with a buffer containing 16.7 mM Tris-HCl (pH 8.0), 167 mM NaCl, 1.2 mM EDTA, and 1.1% Triton-X, fragmented chromatins were immunoprecipitated with KLF6 antibody or normal rabbit IgG (Santa Cruz Biotechnology, Inc.) at 4°C. Immune complexes were washed with washing buffer (20 mM Tris-HCl (pH 8.0), 150 mM NaCl, 2 mM EDTA, 1% Triton-X, and 0.1% SDS) and further incubated with protease K (Fermentas, Hanover, MD, USA) at 55°C. Isolated DNAs were precipitated and analyzed by PCR using specific primers (Figure S5B).

**Statistical analysis**

Data are expressed as mean ± SEM of three independent experiments. The statistical significance of the data was analyzed via Student’s t-test (n.s, not significant with *p* > 0.05, *, *p* < 0.05, **, *p* < 0.01, ***, *p* < 0.001).

**Data availability**

The data used and analyzed during the current study are available within the manuscript and its additional files. Additional data are available from the corresponding author upon reasonable request.

**Reference**

1. Schneider CA, Rasband WS, Eliceiri KW. NIH Image to ImageJ: 25 years of image analysis. *Nat Methods.* 2012;9(7):671-675.
